# Microstructure & physicochemical properties dataset of NaCl-based salt mixtures for concentrating solar power

**DOI:** 10.1038/s41597-025-06437-z

**Published:** 2026-01-08

**Authors:** Yun Feng, Yang Wu, Wenhao Wang

**Affiliations:** 1 School of Materials & Metallurgy, Guizhou University, Guiyang, 550025 China; 2 Beijing Key Laboratory of Process Automation in Mining & Metallurgy, State Key Laboratory of Intelligent Optimized Manufacturing in Mining & Metallurgy Process, Beijing, 102628 China; 3 Zunyi Titanium Co., Ltd, Zunyi, 563204 China

**Keywords:** Theory and computation, Chemical physics

## Abstract

Concentrating solar power is a pivotal technology in global transition toward renewable energy, providing a viable pathway for dispatchable and base-load electricity generation. An important component of the concentrating solar power system is molten salts, particularly NaCl-based mixtures, which serve as both efficient heat transfer fluids and high-capacity thermal energy storage media. The influence mechanisms of micro-ionic interactions and microstructure on physicochemical properties of NaCl-based molten salt mixtures play a decisive role in exploration of more efficient molten salt formulations. We present a dataset of microstructure and physicochemical properties of NaCl-based molten salt mixtures for concentrating solar power, which involves thermal expansion coefficient, thermal conductivity, specific enthalpy of fusion, specific heat capacity, density, and viscosity of mixtures, ionic self-diffusion coefficient, coordination bond angle and coordination bond length of ion pairs, and coordination number of ions across varying elemental compositions and a wide temperature ranges from 556 K to 1400 K, which significantly exceeds the current operating limits of commercial nitrate-based solar salt. The dataset may help to integrate concentrating solar power with other renewable energy technologies, which is essential for maximizing its impact on global climate change mitigation efforts.

## Background & Summary

Concentrating solar power (CSP) coupled with thermal energy storage (TES) has attracted significant attention in recent decades as a promising renewable energy technology^1,2^. CSP systems generate electricity by concentrating sunlight to heat molten salt, which serves as both an efficient heat transfer fluids and a high-capacity thermal energy storage media, and subsequently converts thermal energy into electrical power^3,4^. Currently, sodium nitrate (NaNO_3_) and potassium nitrate (KNO_3_), known for their high energy densities and low vapor pressures, are typically blended in a 60:40 mass ratio to form the commercially available “*solar salt*”^5,6^. This binary nitrate mixture has become one of the most widely used heat transfer fluids and thermal energy storage media in CSP plants. Although compatible with the operating range of existing steam turbines, these commercial nitrate salt mixtures face thermal stability challenges, as they begin to decompose when operating temperatures exceed 823 K^7,8^.

To increase the thermal energy efficiency of CSP systems, which is fundamentally governed by the Carnot principle, operation at higher temperatures is essential. This drives the exploration of alternative molten salts with superior thermal stability. Among these, NaCl-based molten salt mixtures, such as NaCl-MgCl₂^9,10^, NaCl-CaCl₂^11,12^, and NaCl-KCl-MgCl₂-LaCl₃^13^, have emerged as highly promising candidates for next-generation TES applications. These chloride-based systems exhibit superior thermal stability and thermal energy storage potential at elevated temperatures, significantly exceeding the thermal stability limit of conventional nitrate-based solar salt (~823 K). For instance, the thermal conductivity of the mixtures typically ranges from 0.30 to 0.60 W·m^−1^·K^−1^ across a wide temperature range, from 1000 K to 1500 K, highlighting their enhanced high-temperature performance. However, a key challenge in optimizing these materials lies in understanding the fundamental relationships between their microscopic ionic interactions, microstructure, and macroscopic physicochemical properties^4,14^. Experimental characterization of molten salts at extreme temperatures is often limited by technical constraints, leading to sparse and sometimes inconsistent data. To address this gap, computational approaches, including the molecular dynamics simulations^15^ and the *ab*-initio calculations^16^, have become indispensable tools for elucidating the behaviour of molten salts and guiding the development of advanced TES materials.

This work consolidates and analyses published data on NaCl-based molten salt mixtures, providing a dataset on their microstructure and physicochemical properties. The dataset includes thermal expansion coefficient, thermal conductivity, specific enthalpy of fusion, specific heat capacity, density, and viscosity of mixtures, ionic self-diffusion coefficient, coordination bond angle and coordination bond length of ion pairs, and coordination number of ions across varying elemental compositions and temperature ranges from 556 K to 1400 K. The compiled information serves as a valuable resource for researchers and engineers, facilitating data-driven material design, machine learning model training, and the development of optimized molten salt formulations for advanced thermal storage applications. Furthermore, this work supports the integration of CSP industries with other renewable energy technologies, a critical step toward maximizing its role in global climate change mitigation efforts.

## Methods

### Boundary definition and research strategies

We conducted a systematic and comprehensive public publication search to identify relevant studies on the NaCl-based molten salt mixtures. The Web of Science database (https://webofscience.clarivate.cn/wos/alldb/basic-search) was selected as the primary search platform due to its extensive coverage of high-quality scientific publications. Academic interest in the microstructure and physicochemical properties of molten salt mixtures for the CSP was notably lacking prior to 2015^14,17^. This search was restricted to Article and Review published from 2015-01-01 to 2025-03-31, to focus on recent advances in computational and experimental methods for the molten salt mixtures.

The search strategy employed a combination of keywords, including *“NaCl-based molten salts”, “high-temperature molten salts”, “molecular dynamics simulation”, “first-principles calculation”*, and *“computational thermodynamics”* to ensure broad retrieval of potentially relevant studies. The backward citation tracking, examining references of the key publications, and forward citation tracking, identifying newer studies citing the retrieved works, were employed to identify the additional relevant publications, to minimize selection bias and to capture important contributions that might have been missed in the initial database search.

**Fig. 1:**
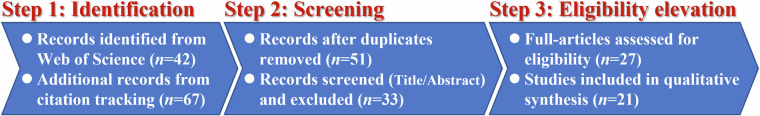
Flowchart of the literature screening and selection process.

The indexed publications were first filtered through the title and abstract relevance screening to identify studies providing both sufficient methodological details and quantitative data on either microstructure parameters or physicochemical properties of NaCl-based molten salt mixtures. And then, the publications with extractable and reproducible results were further filtered by the full-text evaluation. With this rigorous screening process, as shown in Fig. 1, only 21 high-quality publications, detailed in Table 1, were retained for in-depth analysis.

**Table 1:** Details of remaining 21 available publications for in-depth analysis.

No.	Year	Reference DOI
1	2015	10.1016/j.molliq.2015.06.021^15^
2	2017	10.1016/j.molliq.2016.12.091^25^
3	2017	10.1016/j.nanoen.2017.07.020^26^
4	2018	10.1016/j.molliq.2017.11.068^27^
5	2020	10.1016/j.solener.2020.09.038^28^
6	2020	10.1016/j.solmat.2020.110696^16^
7	2020	10.1016/j.solmat.2020.110504^29^
8	2021	10.1016/j.renene.2020.08.152^12^
9	2021	10.1016/j.solmat.2021.111351^30^
10	2021	10.1016/j.molliq.2021.117321^31^
11	2022	10.1016/j.jnucmat.2022.153916^9^
12	2022	10.1016/j.est.2022.104707^32^
13	2022	10.1016/j.molliq.2021.117054^33^
14	2023	10.1021/acsami.3c13412^34^
15	2023	10.1021/acsami.2c19272^10^
16	2023	10.1039/d3ta03434h^13^
17	2023	10.1002/adts.202200833^35^
18	2023	10.1016/j.solmat.2022.112108^36^
19	2024	10.1016/j.solmat.2024.113091^37^
20	2024	10.1016/j.solmat.2024.112903^38^
21	2024	10.1007/s11630-024-2054-5^11^

### Data collection and management

**Fig. 2:**
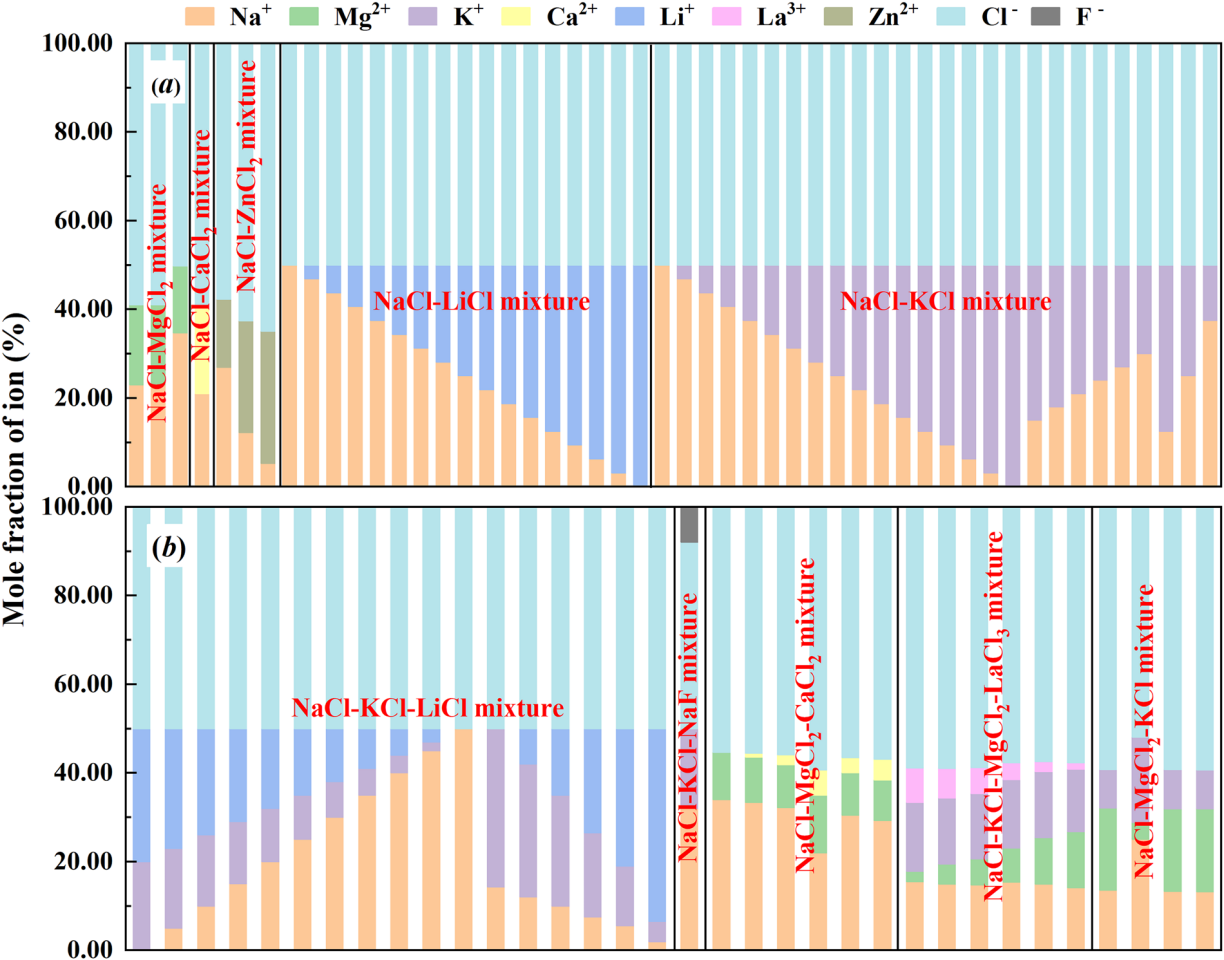
Elemental composition of (**a**) binary mixtures and (**b**) ternary & quaternary mixtures in present dataset.

The NaCl-based molten salt mixtures in this dataset encompass a variety of compositions, including the binary mixtures, such as NaCl-MgCl_2_ salt, NaCl-CaCl_2_ salt, NaCl-LiCl salt, NaCl-KCl salt, and NaCl-ZnCl_2_ salt, the ternary mixtures, like NaCl-MgCl_2_-KCl salt, NaCl-MgCl_2_-CaCl_2_ salt, NaCl-KCl-NaF salt, and NaCl-KCl-LiCl salt, as well as the quaternary mixture, such as NaCl-KCl-MgCl_2_-LaCl_3_ salt. The elemental composition of the above-mentioned NaCl-based molten salt mixtures is systematically summarized in Fig. 2, providing a clear overview of their ionic constituents.

The computational or experimental results in the publications for the molten salt mixtures are typically presented in two formats: tabular data and graphical representations. Numerical values listed in tables can be directly extracted for analysis, whereas data presented in figures often require digitization to ensure the data can be further processed. For this purpose, tools such as WebPlotDigitize*r* (https://plotdigitizer.com) are widely used to accurately extract numerical values from plotted curves or scatter points.

**Fig. 3:**
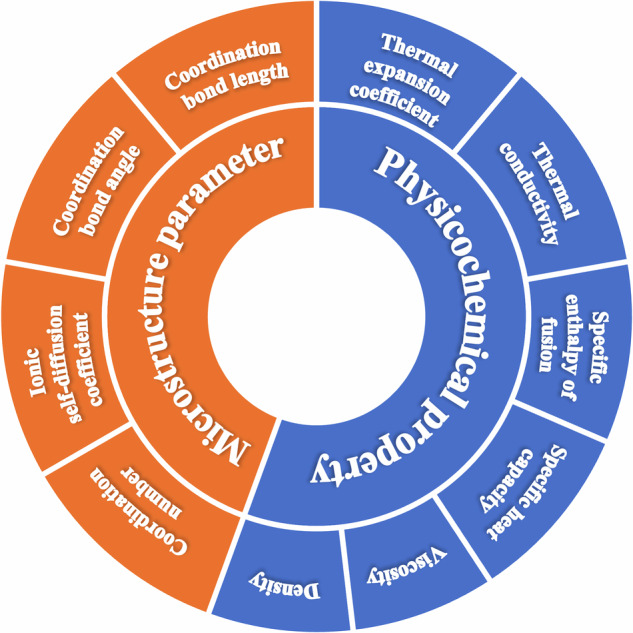
Classification of available data extracted from the publications.

**Fig. 4:**
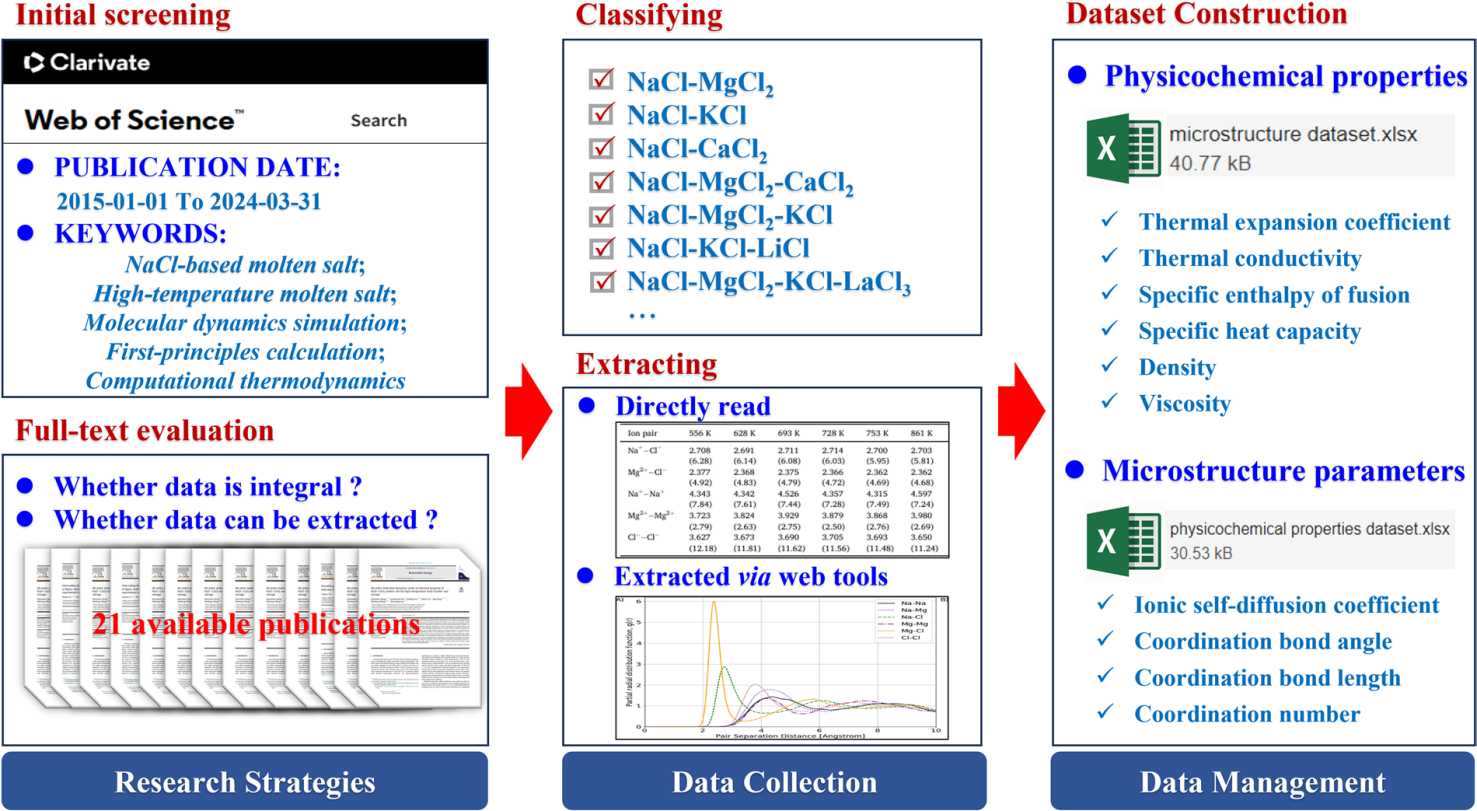
Flowchart of entire processing procedure of the present dataset.

We systemically extracted available data from the publications, including the thermal expansion coefficients, thermal conductivity, specific enthalpy of fusion, specific heat capacity, density, and viscosity of the molten salt mixtures, the ionic self-diffusion coefficient, the coordination bond angle and coordination bond length of the ion pairs, and the coordination number of ions, with various elemental compositions in different temperature ranges. The explicit definitions and units of those parameters for molten salt mixtures were provided in Table 2. And two distinct datasets were meticulously compiled to facilitate: the physicochemical properties dataset and the microstructure dataset, which illustrated in Fig. 3. It is important to note that certain data records may exhibit missing parameters due to limitations in the source literature, including instances where specific properties were not computed or reported incompletely. The entire processing work of the present dataset is described in Fig. 4.

**Table 2:** Explicit definitions and units of parameters for molten salt mixtures.

Name	Symbol	Unit	Definition
Elemental composition			Atomic species and quantity of the molten salt mixtures
Temperature	*T*	K	Temperature used in experiment or calculation, a fundamental variable influencing material properties
Thermal expansion coefficient	*β*	K^−1^	Dimensional change rate under thermal stress
Thermal conductivity	*λ*	W·m^−1^·K^−1^	Ratio of heat flux density and temperature gradient to reflecting heat transfer efficiency
Specific enthalpy of fusion	*ΔH*_*f*_	J·g^−1^	Latent thermal energy intensity of solid-fusion phase change
Specific heat capacity	*c*	J·g^−1^·K^−1^	Thermal energy required to raise the temperature by 1 K of a unit mass
Density	*ρ*	g·cm^−3^	Mass of molten salt per unit volume
Viscosity	*η*	Pa·s	Ratio of shear stress and velocity gradient of fluid to reflect internal friction resistance to fluid flow
Ionic self-diffusion coefficient	*D*	m^2^·s^−1^	Ionic mobility within the molten salt
Coordination bond angle	*θ*	°	Local symmetry and geometrical bonding of the ion pairs that calculated from angular distribution function
Coordination bond length	*r*	Å	Length of ionic bonds determined *via* the radial distribution function
Coordination number			Average value of directly coordinated ions

The relative uncertainties of those parameter data are below 1.0%, as reported in the referenced publications^18,19^.

#### Data Records

Two Excel-based datasets were created and are presented in Table 3. The details of the thermal expansion coefficient, thermal conductivity, specific enthalpy of fusion, specific heat capacity, density, and viscosity of the NaCl-based molten salt mixtures in different temperature ranges are recorded in an Excel file named *physicochemical properties dataset*. And another Excel file named *microstructure parameters dataset* contains the details of the ionic self-diffusion coefficient, coordination bond angle, coordination bond length, and coordination number of different ion pairs for the mixtures in different temperature ranges. The database was uploaded and publicly available at the *Figshare* repository^20^ and is available for download in Excel format.

**Table 3:** Overview of two Excel files in present dataset.

Number	Name	Details
1	physicochemical properties dataset	Details of the thermal expansion coefficient, thermal conductivity, specific enthalpy of fusion, specific heat capacity, density, and viscosity of NaCl-based molten salt mixtures in different temperature ranges
2	microstructure parameters dataset	Details of the ionic self-diffusion coefficient, coordination bond angle, coordination bond length, and coordination number of different ion pairs of NaCl-based molten salt mixtures in different temperature ranges

## Technical Validation

### Physicochemical properties *vs*. temperature of the NaCl-based molten salt mixtures

**Fig. 5:**
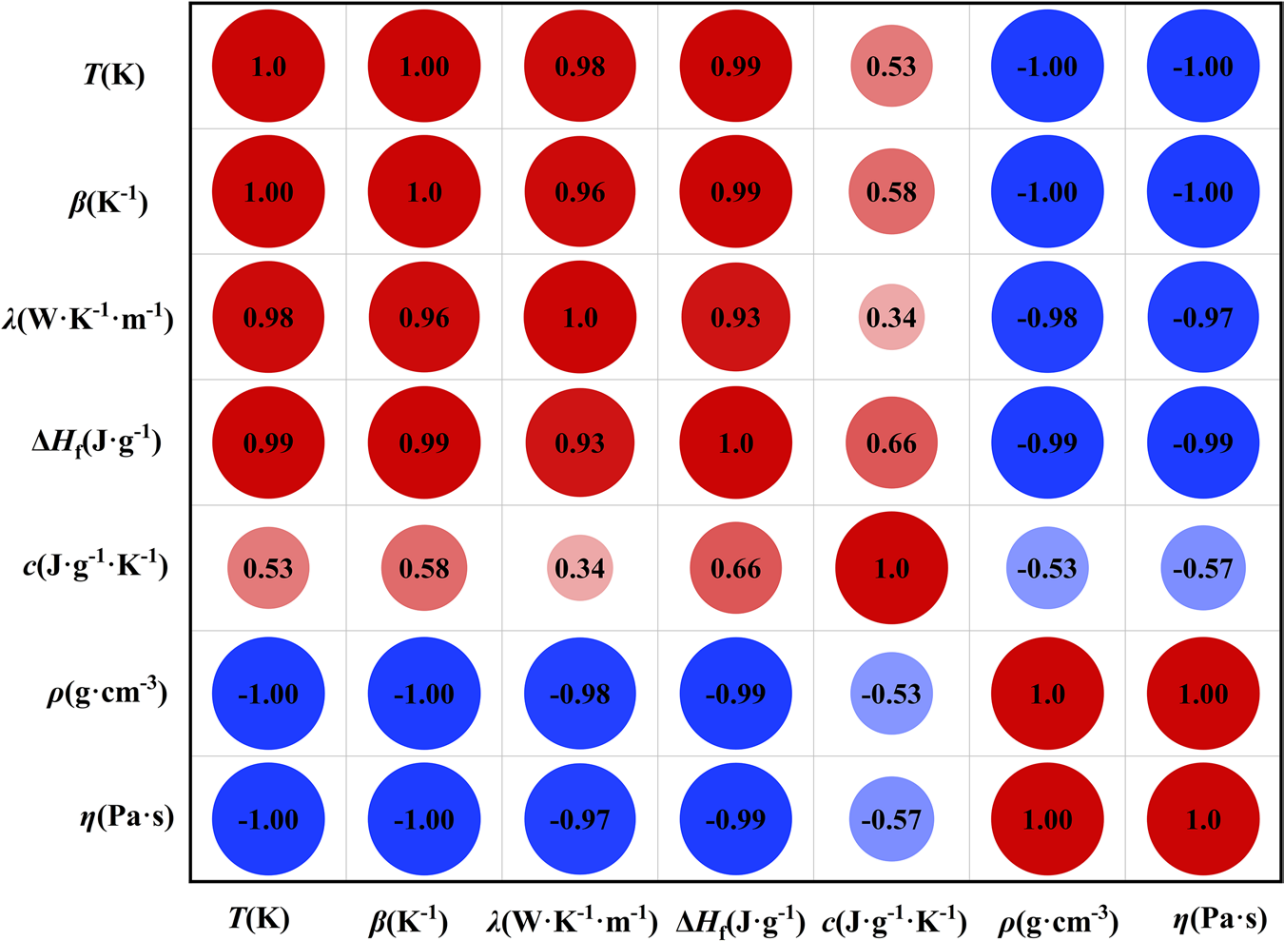
Correlations heatmap of physicochemical properties *vs*. temperature of NaCl-based molten salt mixtures from 556 K to 1400 K.

A Pearson correlation analysis was conducted in this dataset, accompanied by a heatmap to display the correlations between the physicochemical properties *vs*. temperature of the NaCl-based molten salt mixtures, as shown in Fig. 5. The heatmap analysis reveals that the thermal expansion coefficient, thermal conductivity, and specific enthalpy of fusion exhibit significantly positive temperature dependence with higher Pearson correlation coefficients of r = 1.00, r = 0.98, and r = 0.99, respectively. In contrast, density and viscosity display significant inverse correlations with temperature with negative Pearson correlation coefficients r = −1.00 and r = −1.00, respectively^21^. The heat capacity shows moderate positive correlation with *an* intermediate Pearson correlation coefficient r = 0.53.

### Ionic self-diffusion coefficient *vs*. temperature of the NaCl-based molten salt mixtures

**Fig. 6:**
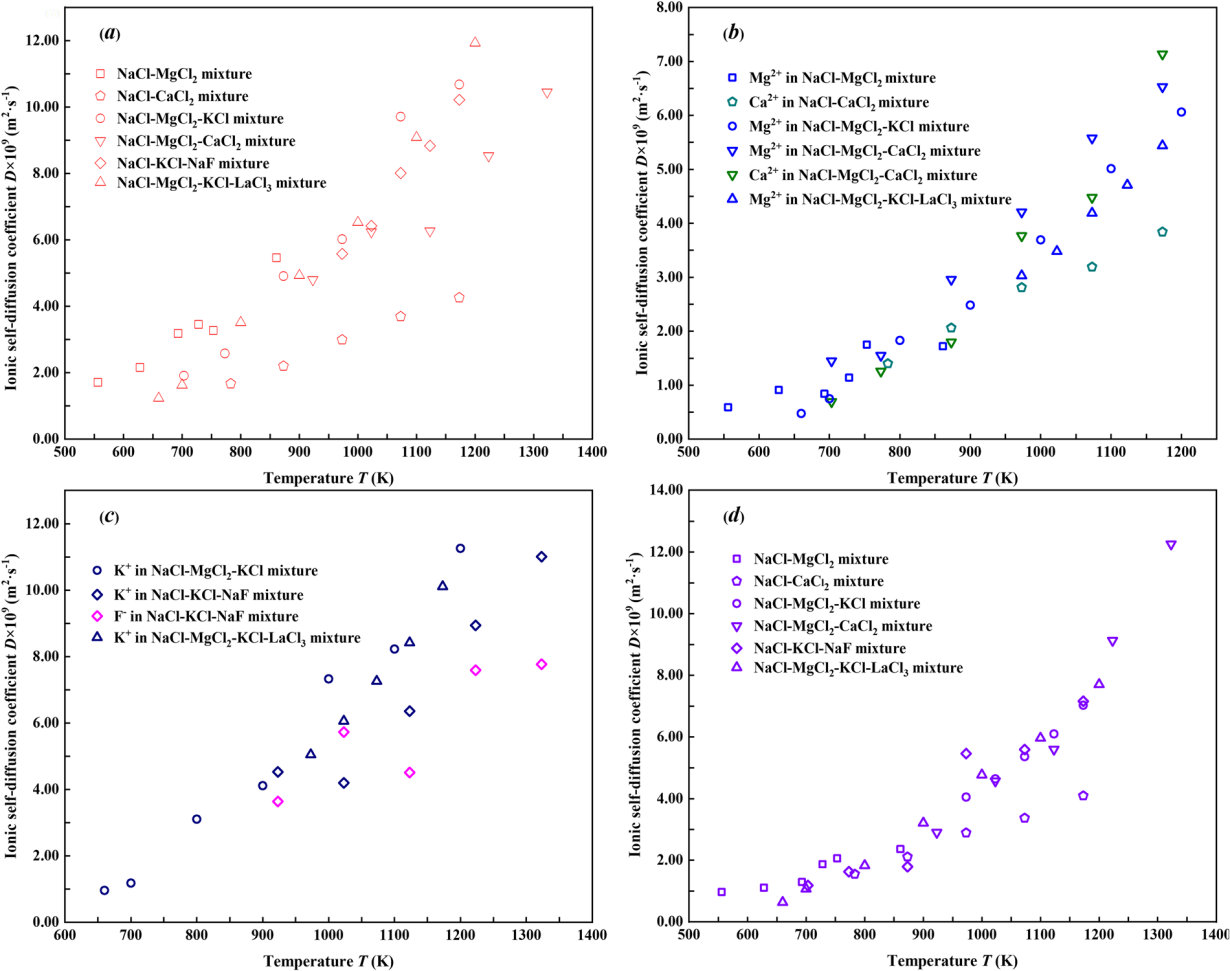
Ionic self-diffusion coefficients of (**a**) Na^+^, (**b**) Mg^2+^ and Ca^2+^, (**c**) K^+^ and F^-^, and (**d**) Cl^-^ in molten salt mixtures from 556 K to 1400 K.

The ionic self-diffusion coefficients could be calculated *via* the mean-squared displacement method^22^. Consistent with fundamental thermodynamic and diffusion principles, the elevated temperatures promote ionic thermal agitation, resulting in a monotonic increase in the ionic self-diffusion coefficients with increasing temperature in Fig. 6, which illustrates the temperature dependence for the ionic self-diffusion coefficients of Na^+^, Mg^2+^, Ca^2+^, K^+^, F^-^, and Cl^-^ in molten salt mixtures, respectively. The trends not only align with theoretical expectations but also demonstrate the internal consistency and thermodynamics validity in the present dataset.

### Coordination bond length of ion pairs *vs*. temperature of the NaCl-based molten salt mixtures

**Fig. 7:**
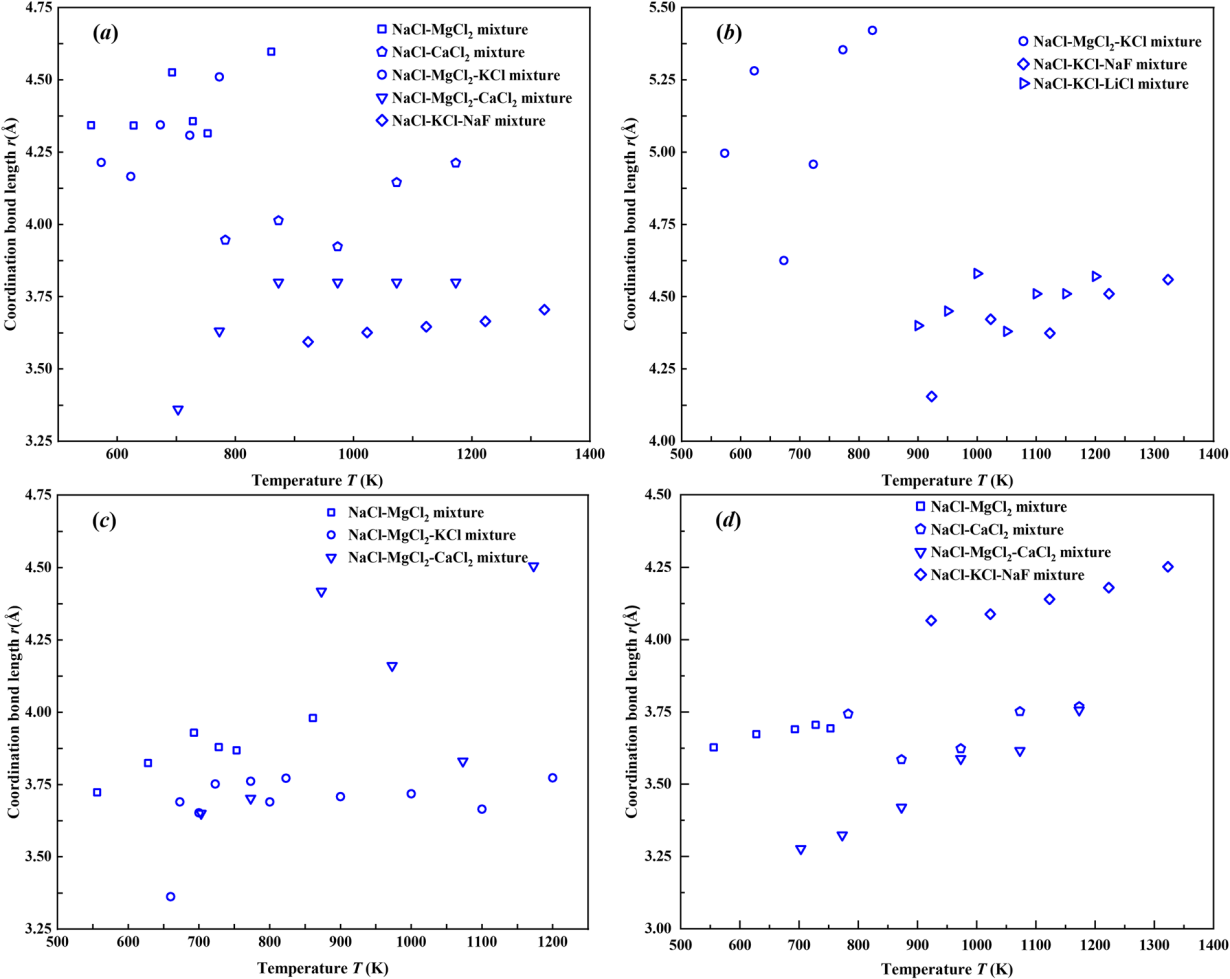
Coordination bond lengths of like-charged ion pairs (**a**) Na^+^-Na^+^, (**b**) K^+^-K^+^, (**c**) Mg^2+^-Mg^2+^, and (**d**) Cl^–^Cl^-^ in molten salt mixtures from 556 K to 1400 K.

**Fig. 8:**
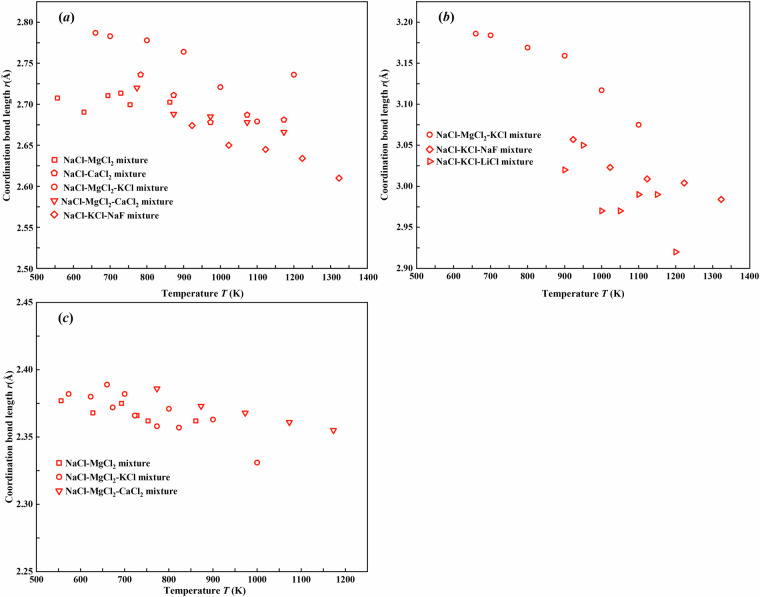
Coordination bond lengths of oppositely-charged ion pairs (**a**) Na^+^-Cl^-^, (**b**) K^+^-Cl^-^, and (**c**) Mg^2+^-Cl^-^ in molten salt mixtures from 556 K to 1400 K.

The coordination bond length of ion pairs servers as a crucial parameter for characterizing the microstructure of the molten salt mixtures^23,24^. These bond lengths are typically determined through the radial distribution function analysis in molecular calculations. Figures 7, 8 present the temperature dependence of the coordination bond lengths for different ion pairs from 556 K to 1400 K. Notably, the cation-cation pairs (Na^+^-Na^+^, K^+^-K^+^, and Mg^2+^-Mg^2+^) and the anion-anion pairs (Cl^–^Cl^-^) exhibit consistent decrease trends in the coordination bond lengths with increasing temperature, as shown in Fig. 7. All examined cation-anion pairs (Na^+^-Cl^-^, K^+^-Cl^-^, and Mg^2+^-Cl^-^) demonstrate opposite trends in Fig. 8, showing gradual elongation of the coordination bond lengths at elevated temperature. These two contrasting trends suggest fundamentally different temperature-dependent interactions between like-charged and oppositely-charged ions in the NaCl-based molten salt mixtures.

## Usage Note

This dataset serves as a critical resource for researchers and engineers in material screening and design. It enables the rapid identification of promising NaCl-based or MgCl₂-based molten salt compositions with desired thermophysical properties for next-generation CSP plants. Concurrently, it functions as a benchmark for validating the molecular dynamics and the *ab*-initio simulations, as well as a high-quality training dataset for developing machine learning models in materials informatics. Furthermore, the dataset provides essential input parameters for system-level modeling and simulation of the CSP thermal energy storage and heat transfer loops.

Although the data is structured in two separate Excel files at the *Figshare* repository to allow for focused analysis, it is recommended that users consult the original publications that provided in Table 1 for more detailed results and to facilitate effective use. The dataset will be periodically updated to include new compositions and properties, maybe not only limited to NaCl-based mixtures.

## Data Availability

The dataset can be downloaded online directly from the repository: 10.6084/m9.figshare.28869017.v4.

## Code availability

There was no code used in the generation of the data in this work, an only Microsoft Excel is employed to process all the data. And the data in all figures were extracted by Web Plot Digitizer (https://plotdigitizer.com).
